# Incidence, Seasonality and Mortality Associated with Influenza Pneumonia in Thailand: 2005–2008

**DOI:** 10.1371/journal.pone.0007776

**Published:** 2009-11-11

**Authors:** James Mark Simmerman, Malinee Chittaganpitch, Jens Levy, Somrak Chantra, Susan Maloney, Timothy Uyeki, Peera Areerat, Somsak Thamthitiwat, Sonja J. Olsen, Alicia Fry, Kumnuan Ungchusak, Henry C. Baggett, Supamit Chunsuttiwat

**Affiliations:** 1 International Emerging Infections Program, Thailand Ministry of Public Health–United States Centers for Disease Control Collaboration, Nonthaburi, Thailand; 2 Influenza Division, United States Centers for Disease Control and Prevention, Atlanta, Georgia, United States of America; 3 Thailand Ministry of Public Health, Nonthaburi, Thailand; 4 Division of Emerging Infections and Surveillance Services, Centers for Disease Control, Atlanta, Georgia, United States of America; University of Cape Town, South Africa

## Abstract

**Background:**

Data on the incidence, seasonality and mortality associated with influenza in subtropical low and middle income countries are limited. Prospective data from multiple years are needed to develop vaccine policy and treatment guidelines, and improve pandemic preparedness.

**Methods:**

During January 2005 through December 2008, we used an active, population-based surveillance system to prospectively identify hospitalized pneumonia cases with influenza confirmed by reverse transcriptase–polymerase chain reaction or cell culture in 20 hospitals in two provinces in Thailand. Age-specific incidence was calculated and extrapolated to estimate national annual influenza pneumonia hospital admissions and in-hospital deaths.

**Results:**

Influenza was identified in 1,346 (10.4%) of pneumonia patients of all ages, and 10 influenza pneumonia patients died while in the hospital. 702 (52%) influenza pneumonia patients were less than 15 years of age. The average annual incidence of influenza pneumonia was greatest in children less than 5 years of age (236 per 100,000) and in those age 75 or older (375 per 100,000). During 2005, 2006 and 2008 influenza A virus detection among pneumonia cases peaked during June through October. In 2007 a sharp increase was observed during the months of January through April. Influenza B virus infections did not demonstrate a consistent seasonal pattern. Influenza pneumonia incidence was high in 2005, a year when influenza A(H3N2) subtype virus strains predominated, low in 2006 when A(H1N1) viruses were more common, moderate in 2007 when H3N2 and influenza B co-predominated, and high again in 2008 when influenza B viruses were most common. During 2005–2008, influenza pneumonia resulted in an estimated annual average 36,413 hospital admissions and 322 in-hospital pneumonia deaths in Thailand.

**Conclusion:**

Influenza virus infection is an important cause of hospitalized pneumonia in Thailand. Young children and the elderly are most affected and in-hospital deaths are more common than previously appreciated. Influenza occurs year-round and tends to follow a bimodal seasonal pattern with substantial variability. The disease burden varies significantly from year to year. Our findings support a recent Thailand Ministry of Public Health (MOPH) decision to extend annual influenza vaccination to older adults and suggest that children should also be targeted for routine vaccination.

## Introduction

Influenza is a common vaccine preventable viral infection that can cause severe or fatal disease in the elderly, the very young and those with underlying illness [1], [2], [3], [4], [5]. In temperate climates of Europe and North America, wintertime seasonal influenza epidemics often result in dramatic increases in hospitalization, death and significant economic losses due to workplace absenteeism [4], [6], [7]. Much less is known about the burden of influenza morbidity and mortality in tropical and subtropical countries [8], [9]. Data from Hong Kong and Singapore suggest that influenza is an important cause of illness with rates of hospitalization and mortality comparable to that of the United States [10], [11], [12], [13]. The contribution of influenza virus infection to pneumonia, the leading cause of pediatric mortality, has also not been determined. This question is particularly important for developing countries in tropical and subtropical climates where most childhood pneumonia occurs and where influenza has historically been perceived as a mild or uncommon disease. Prospective, multi-year, population-based data on laboratory-confirmed influenza infection are needed to describe the disease burden and inform prevention and control strategies during both interpandemic and pandemic periods.

Thailand is a middle-income country with a 2005 population of 65,112,652 million and a well-developed public health system[14]. The climate is subtropical with monsoon rains during the months of May to October. Since 2003, national pandemic planning and an improved understanding of influenza epidemiology have increased interest in influenza vaccination. A 2004 study by Simmerman and colleagues concluded that influenza infection is associated with substantial health care and social costs in Thailand[15]. Since 2004, the Thailand Ministry of Public Health (MOPH) has offered free influenza vaccination to approximately 400,000 health care workers each year. In 2007 this coverage was extended to persons 65 years of age and older with underlying risk factors and development began on a domestic influenza vaccine production facility [16], [17]. In 2000, 72,102 influenza vaccine doses were distributed; 750,000 doses were distributed in 2006; 1.3 million doses were distributed in 2008, and approximately 2.4 million doses were distributed in the public and private sectors in 2009 [18], [19]. While this increase in vaccine uptake is notable, in 2009 influenza vaccine coverage was less than 4% across the national population The expansion of the influenza vaccination program may have also improved Thailand's ability to respond to the 2009 H1N1 pandemic by expanding national immunization program capacity and experience with seasonal vaccination campaigns [20]. Additional data from multiple years are needed to guide decisions on the expansion of influenza vaccination in Thailand and neighboring countries. We describe the incidence, seasonality and in-hospital mortality associated with hospitalized influenza pneumonia from two provinces in Thailand during four consecutive years and extrapolate our findings to provide national estimates of disease burden.

## Methods

**Figure 1:**
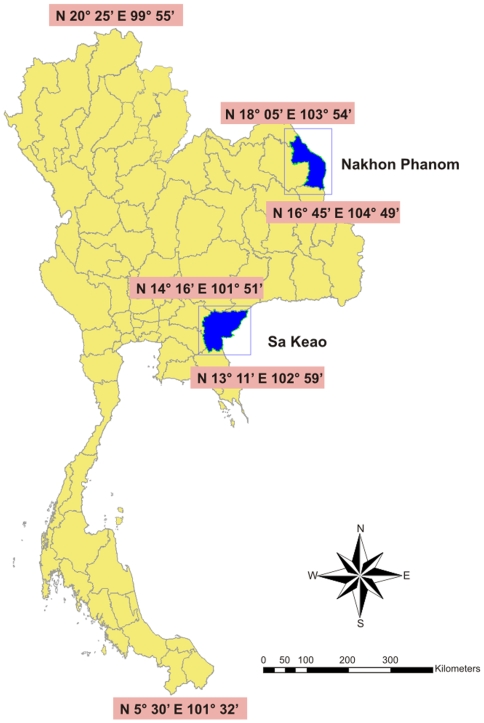
Sa Kaeo and Nakhon Phanom Provinces in Thailand.

We prospectively identified all hospitalized pneumonia patients during January 2005 through December 2008 using an active, population-based surveillance system carried out through collaboration between the Thailand MOPH and the U.S. Centers for Disease Control and Prevention (CDC) in Sa Kaeo province in eastern Thailand and Nakhon Phanom province in northeast Thailand (Figure 1)[21], [22] . Sa Kaeo (pop 526,432) and Nakhon Phanom (pop 734,000) are rural provinces with age distributions that are similar to the national population [23]. The 2007 population densities of Sa Kaeo and Nakhon Phanom were 75 and 126 persons per km^2^ respectively, compared to 111 per km^2^ for all of Thailand outside of Bangkok. The same year among 76 Thai provinces, Nakhon Phanom ranked 74^th^ with a per capita GDP of $891 while Sa Kaeo ranked 50^th^ with a per capita GDP of $1,632 (national range $858–$30,457 USD) [24]. During 2008 the mean daily temperature in Sa Kaeo province was 26.8 degrees Celsius with 1602 mm of total rainfall. The daily mean temperature in Nakhon Phanom was 25.4 degrees Celsius and 2786 mm of rainfall was recorded. For comparison, Thailand's mean daily temperature was 26.9 degrees and an average of 914.7 mm of rain fell during 2008 [25].

**Figure 2:**
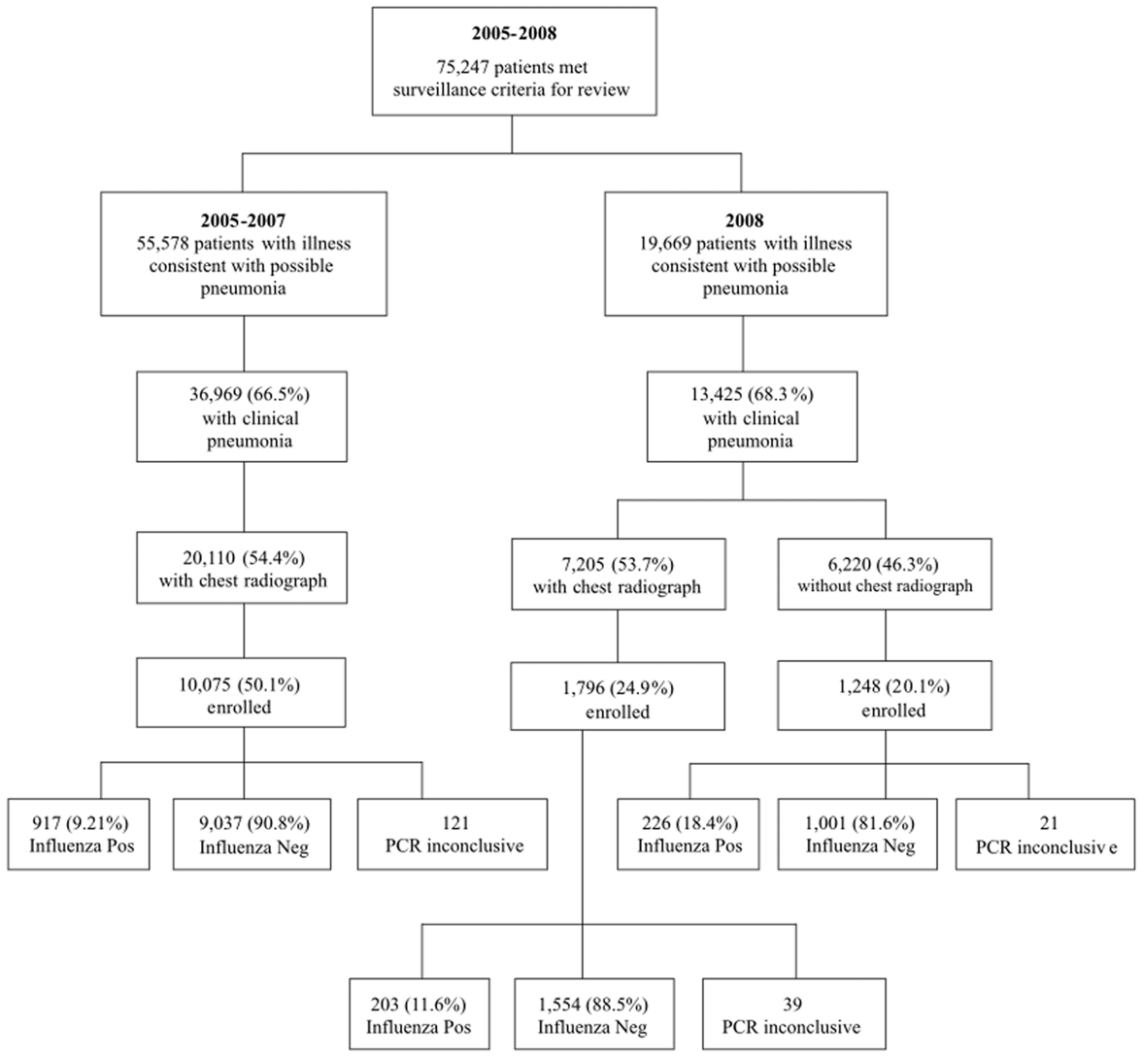
Enrollment process.

As part of a larger prospective study of pneumonia etiology, patients with clinical pneumonia were enrolled from all 20 hospitals in both provinces [22], [26]. A case of clinical pneumonia was defined in patients with evidence of both acute infection (either reported fever, reported chills, measured temperature >38.2 or <35°C, or an abnormal white blood cell count or differential) and lower respiratory tract symptoms (abnormal breath sounds, tachypnea, cough, sputum production, or dyspnea). From January 1, 2005 to Dec 31, 2007 patients with clinical pneumonia and a chest radiograph taken within 48 hours of admission were eligible for enrollment. Chest radiographs were later digitized and read by a panel of radiologists in Bangkok [27]. Beginning in January 2008, the requirement for a chest radiograph for study enrollment was dropped. To limit study costs and laboratory workload resulting from the expanded eligibility criteria, every other patient with clinical pneumonia was approached for enrollment (Figure 2).

Patients transferred from another hospital, readmitted within three days from the same hospital, and newborn infants were excluded to avoid recording nosocomial infections. Patients who were readmitted and reenrolled within 14 days were excluded from the analysis. A nasopharyngeal swab specimen was collected within 48 hours of admission after informed written consent was obtained. The specimen was frozen at −70 degrees Celsius until tested at the Thai National Institute of Health (NIH) laboratory by reverse transcriptase–polymerase chain reaction (RT-PCR) (2005–2008) and cell culture (only during 2005) using Madine-Darby Canine Kidney cells using procedures provided by the Centers for Disease Control and Prevention, U.S.A. (Stephen Lindstrom, personal communication). Specific protocols are available upon request from CDC [28], [29]. A systematic, blinded laboratory quality control system was instituted to monitor diagnostic accuracy in coordination with the influenza laboratories at the US CDC in Atlanta, Georgia. Data were entered into a database (Microsoft Access) and statistical analyses were conducted using Microsoft Excel and SAS version 9.1 (Cary, NC).

The age-specific incidence of laboratory-confirmed influenza pneumonia was calculated by dividing the total number of influenza cases among enrolled patients in each age category by the combined corresponding age-specific population of both provinces and is hereafter referred to as the minimum incidence. We then calculated age-specific incidence adjusted for patients who were eligible but who were not enrolled by applying the same rate of influenza positivity as measured in each age category in the enrolled group. To account for the different eligibility criteria in 2008, we adjusted the age-specific influenza incidence among those with a chest radiograph and multiplied by two to account for the 50% sampling frame. We applied a chi squared test of proportion to determine if study participants with or without a chest radiograph were more likely to have influenza pneumonia during 2008. Next, we estimated the number of influenza pneumonia hospitalizations nationwide by applying the minimum and adjusted incidence from the two study provinces to the Thai population using 2006 national census figures. We used a similar approach for in-hospital mortality. We grouped these relatively rare fatal events into three age categories (<15, 15–50, >50) and then estimated national in-hospital influenza-associated mortality. The median length of hospital stay for influenza pneumonia was determined using data from all cases. The seasonal distribution of influenza activity was plotted as a moving average of the monthly proportion of influenza positive patients among all pneumonia patients enrolled in the study.

## Results

From January 2005 through December 2007, we enrolled 10,075 (50.1%) of 20,100 patients. Specifically, we enrolled 3,292 patients in 2005, 3,633 patients in 2006 and 3,150 patients in 2007. In 2008 when chest radiographs were no longer required for eligibility, 13,425 patients were eligible and 50% were invited to participate. Among 7,205 patients with a chest radiograph, 1,796 (24.9%) were enrolled and among 6,220 without a chest radiograph, 1,248 (20.1%) were enrolled (Figure 2). Overall, 1,346 (10.3%) of enrolled clinical pneumonia patients tested positive for influenza by RT-PCR or cell culture. Among the 1,346 pneumonia patients with influenza, 707 (52.5%) had results evaluated by the radiologist panel. Four hundred and eight of 670 (60.9%; 37 missing) had evidence of pneumonia, including 40 of 387(5.9%; 21 missing) with consolidation and 327 of 393 (83.2%; −15 missing) with interstitial infiltrates.

In 2005, a year when Thailand's National Influenza Center (NIC) reported that influenza A (H3N2) virus strains predominated, 527 (16.0%) of 3,292 enrolled hospitalized pneumonia patients tested positive for influenza. In this single year when both cell culture and RTPCR were used to identify influenza virus, of 146 cell cultures that were influenza positive only 7 were not also PCR positive. In 2006, influenza A (H1N1) virus strains predominated and only 147 (4.1%) of 3,632 patients were influenza positive. In 2007, influenza type B and A (H3N2) viruses were approximately equally represented and 243 (7.7%) of 3,150 patients tested positive for influenza. In 2008 influenza type B predominated and 429 (14.1%) of 3,044 patients tested positive for influenza. In 2008, 203 (11.3%) of 1,796 pneumonia patients with chest radiographs were influenza positive compared to 220 (18.1%) of 1,214 patients who had not received a chest radiograph (p<0.0001).

**Figure 3:**
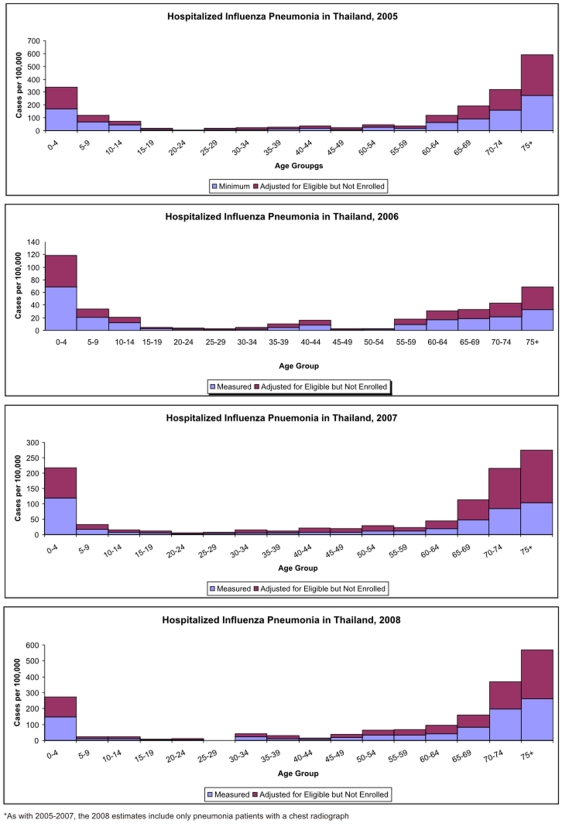
Age-specific incidence of influenza pneumonia 2005. (n = 534), 2006 (n = 147), 2007 (n = 243), 2008 (n = 203).

The minimum incidence of hospitalized influenza pneumonia with chest radiograph among all age groups was 42.1 per 100,000 persons in 2005, 11.7 per 100,000 in 2006, 19.1 per 100,000 in 2007 and 32.7 per 100,000 in 2008. In 2008, when patients both with and without a chest radiograph were included, the minimum incidence was 67 per 100,000. Children under 5 years of age and adults over 65 years of age were most affected in each year. When we adjusted for patients with a chest radiograph who were eligible but were not enrolled, the estimated incidence of hospitalized influenza pneumonia was to 83.3 per 100,000 persons in 2005, 21.7 per 100,000 in 2006, 41.6 per 100,000 in 2007, and 65.5 per 100,000 in 2008 (Figure 3). In 2008, when patients both with and without a chest radiograph were included, the minimum incidence was 134.4 per 100,000. When these incidences are applied to Thailand's national population, we estimate that nationwide at least 73,413 and as many as 145,649 hospital admissions for laboratory confirmed influenza pneumonia occurred between January 2005 and December 2008 (Table 1).

**Table 1:** Estimated Annual Influenza Pneumonia Hospital Admissions and In-Hospital Deaths in Thailand, 2005-2008.

YEAR	<15 years		15–50 years		50+ years		Minimum Total		Adjusted Total	
	Admissions	Deaths	Admissions	Deaths	Admissions	Deaths	Admissions	Deaths	Admissions	Deaths
2008^2^	7,833	22	3,675	18	12,205	197	23,713	237	47,196	481
2007^1^	6,761	19	1,886	9	4,993	81	13,640	109	29,609	257
2006^1^	4,897	14	1,156	6	1,839	30	7,892	49	14,209	92
2005^1^	13,597	39	4,035	19	10,535	170	28,167	228	54,635	456
Total	33,088	95	10,753	52	29,572	477	73,413	623	145,649	1,287
Average	8,272	27	2,688	10	7,393	119	18,353	159	36,413	322

1 Adjusted for patients who were eligible but who were not enrolled.

2 As with 2005-2007, these estimates include only pneumonia patients with a chest radiograph.

**Figure 4:**
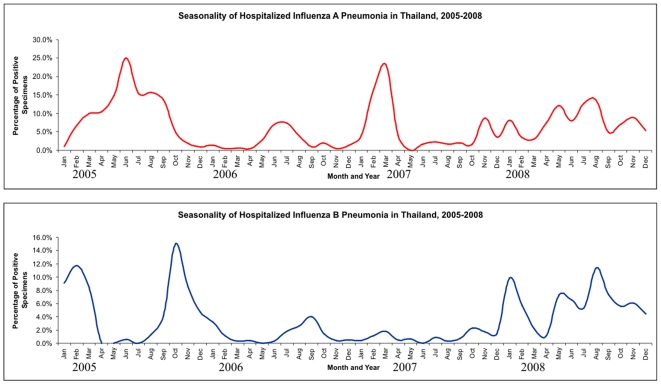
Seasonal distribution of influenza positive pneumonia 2005–2008. (n = 1,346 influenza positive among 13,110 pneumonia cases).

During 2005, 2006 and 2008, pneumonia hospitalizations associated with influenza A virus infections were greatest during the months of June through October. In contrast, in 2007 a sharp increase was observed during the months of January through April. Influenza B virus infections did not demonstrate a consistent seasonal pattern (Figure 4). Among 3,292 pneumonia cases in 2005, influenza A virus infections were identified in 372 (11.3%) patients and influenza B virus infections were identified in 162 (4.9%) patients. In 2006, 92 (2.5%) influenza A virus infections and 55 (1.5%) influenza B virus infections were identified among 3,633 pneumonia cases. In 2007, 210 (6.7%) influenza A virus infections and 33 (1.1%) influenza B virus infections were identified among 3,150 cases. In 2008, 238 (7.8%) influenza A virus infections and 194 (6.4%) influenza B virus infections were identified among 3,044 cases.

The incidence of influenza pneumonia was highest in children less than 5 years of age and in adults greater than 60 years of age during all years. Three hundred and sixty of 4,163 (8%) pneumonia cases in children less than 5 and 117/503 (18.9%) cases in children 5–9 years of age were influenza positive. Seven hundred and two (52%) of all influenza pneumonia patients were less than 15 years of age while 357 (26.5%) were 60 years of age or older. The median length of hospital stay was 4 days for children less than 1 year of age, 4 days for those over 60 years of age, and 4 days (range 0–42 days) across all age groups. Ten (0.75%) case-patients died while in the hospital and 7 of these were in patients older than 50 years of age.

## Discussion

During 2005–2008, across all age groups influenza virus infection was associated with 10.4% of pneumonia cases identified in our study population. These findings extend our 2004 report in which 80 (11%) of 761 pneumonia inpatients had laboratory-confirmed influenza infection [15]. The annual adjusted incidence of hospitalized influenza pneumonia across all age groups was 83, 22, 42 and 66 per 100,000 persons in 2005, 2006, 2007 and 2008, respectively. For comparison, in 2004 we estimated an adjusted incidence of hospitalized influenza pneumonia of 111 per 100,000 across all age groups in Sa Kaeo province [15]. The Thailand MOPH passive surveillance system reported 4.8 cases of clinically diagnosed influenza in hospitalized patients per 100,000 persons in 2005, 4.7 cases per 100,000 in 2006, and 5.7 cases per 100,000 in 2007 [30]. Using U.S. National Hospital Discharge Survey and WHO laboratory data during 1979–2001, Thompson and colleagues estimated that influenza was associated with 8.6% of all primary pneumonia and influenza hospitalizations and the average annual incidence across all age groups was 36.8 per 100,000 (range; 7.8–69.5)[31].

In temperate climates, major variations in influenza activity from year to year have been well-documented [32], [33]. Similarly, substantial year-to-year variability in influenza activity occurs in subtropical Thailand. We observed high levels of influenza activity in 2005, mild activity in 2006, moderate activity in 2007 and high levels of activity in 2008. According to virological surveillance conducted by the Thailand NIC (Malinee Chittaganpitch, personal communication May 2009), influenza A (H3N2) virus strains predominated in 2005. This may explain in part the high levels of influenza pneumonia activity seen in 2005 as influenza A (H3N2) virus strains have been associated with a higher incidence of severe and fatal influenza-attributable disease [34], [35]. In 2006 and 2007 influenza A (H1N1) and influenza type B viruses co-predominated while influenza type B viruses accounted for 50% of all influenza viruses isolated by the Thai NIH during 2008. To our knowledge, this is the first time such marked variability in influenza pneumonia activity has been clearly documented in a subtropical climate and the finding underscores the value of collecting data during several consecutive years.

As observed in 2004, influenza type A activity in Thailand increased sharply during the months of June through October in both 2005 and 2006 [15]. However, this seasonal pattern was not observed in 2007 when a sharp increase in influenza type A activity was observed during January to April. In 2008, both influenza A and B virus activity occurred throughout the year. This finding illustrates the difficulty of effectively timing annual vaccination campaigns in subtropical countries. Currently, vaccination campaigns in Thailand are initiated in April and May using Southern Hemisphere influenza vaccines. Our observations suggest that additional approaches such as instituting routine hospital-discharge influenza vaccination policies for specific groups could be considered.

Our study has limitations that likely resulted in an underestimation of the incidence of influenza pneumonia. Successful identification of influenza virus in clinical specimens depends on factors such as specimen quality, time from illness onset to specimen collection, and optimal transportation and storage of specimens prior to testing. These factors may have contributed to under-ascertainment of influenza pneumonia in our study. Further, we did not attempt to adjust for residents who sought care for pneumonia outside of the study provinces [21], [36] or, during 2005–2007, for pneumonia patients who did not receive a chest radiograph and were therefore excluded from the study. These additional adjustments would likely have substantially increased our incidence estimates.

We observed a nearly four-fold reduction in influenza-positive pneumonia between 2005, a year in which influenza A (H3N2) viruses predominated and 2006 when influenza A (H1N1) viruses predominated. Influenza A (H1N1) infection may be less-often associated with pneumonia. Alternatively, some influenza-positive pneumonia cases may not have felt sufficiently ill to seek care at the hospital. This potential detection bias may have caused us to miss some influenza pneumonia cases and therefore underestimate the disease burden. Finally, after modifying the enrollment criteria in 2008 we observed that patients with clinical pneumonia who had not received a chest radiograph were statistically significantly more likely to be influenza positive. If this was also true during 2005–2007, then our adjustments that assumed the same rates of influenza positivity in both groups (with and without chest radiograph) substantially underestimated the burden of disease.

Our estimates of influenza-associated deaths do not include deaths that were not associated with pneumonia. Thus, our estimates of influenza pneumonia mortality are an underestimation of total influenza-related deaths. Our study was also not designed to ascertain influenza-associated deaths occurring in the community following hospital discharge. Despite these limitations, our estimates of influenza pneumonia in-hospital deaths are much higher than figures reported by the national passive surveillance system. For example, between 1999 and 2006 Thailand reported just four deaths due to human influenza virus infection. This under-ascertainment is likely explained by the lack of laboratory diagnostic capacity for influenza in nearly all public hospitals and to a perception among Thai clinicians that influenza is neither common nor severe.

To calculate national estimates for influenza pneumonia hospitalization, we extrapolated population-based data that captured all pneumonia admissions from our two study provinces (population 1.2 million) under the assumption that the incidence of hospitalized influenza pneumonia does not differ systematically year after year across the 76 provinces of Thailand. While we have no evidence to suggest otherwise, if influenza activity did vary systematically each year by province or geographic region within Thailand, this could affect the accuracy of our national estimates. Influenza associated in-hospital deaths were uncommon and the extrapolation of small numbers to large populations can yield unstable estimates. Regardless, we believe our mortality estimates are conservative and represent an important starting point for further research into this important aspect of disease burden.

Our study contributes to a growing body of published influenza literature from Thailand [15], [18], [36]. Two studies from Thailand reported that influenza virus is an important cause of chronic obstructive pulmonary disease exacerbation and that influenza vaccination is protective in these patients [37], [38]. A 2006 study in Thailand found that 8.6% of children admitted to the national pediatric referral hospital with lower respiratory tract infection or influenza-like illness had cell culture-confirmed influenza virus infection and 80% had no known underlying diseases [39]. A retrospective analysis of risk factors for influenza pneumonia in Thailand found that underlying cardiovascular disease, respiratory conditions and hospital admission in the preceding 12 months were significant risk factors [40]. Finally, a study by Hanshaoworakul and colleagues reported that human influenza was identified in 2,075 (18%) of 11,641 suspected avian influenza A(H5N1) infections with 22 (1%) fatal cases, including seven deaths in children less than ten years of age. In that report, 35% of hospitalized human influenza infections had chest radiograph confirmed pneumonia [41].

In conclusion, we estimated influenza disease burden during four consecutive years using prospective, population-based surveillance and RT-PCR laboratory confirmation in hospitalized pneumonia patients in Thailand. We found evidence of a substantial but variable incidence of influenza pneumonia mainly affecting young children and the elderly. During 2005–2008, influenza pneumonia resulted in an estimated average 36,413 hospital admissions and 322 in-hospital deaths each year. Our findings support the continued development of a national control strategy that extends influenza vaccination to young children and improves access to antiviral medications. Data from other Southeast Asian countries are needed to develop a better understanding of the burden of disease, further describe the contribution of influenza to childhood pneumonia, and to improve regional pandemic preparedness by expanding national immunization program capacities in this region with large populations and rapidly growing economies.
